# Optimizing 802.15.4 Outdoor IoT Sensor Networks for Aerial Data Collection

**DOI:** 10.3390/s19163479

**Published:** 2019-08-09

**Authors:** Michael Nekrasov, Ryan Allen, Irina Artamonova, Elizabeth Belding

**Affiliations:** 1 Department of Computer Science, University of California, Santa Barbara, CA 93106, USA; 2 School of Medicine, the Division of Infectious Diseases and Global Public Health, University of California, San Diego, CA 92093, USA

**Keywords:** Internet of Things, 802.15.4, UAS, UAV, drone, sensor network, wireless networks, aerial networks, experimental measurements

## Abstract

Rural IoT sensor networks, prevalent in environmental monitoring and precision agriculture, commonly operate over some variant of the IEEE 802.15.4 standard. Data collection from these networks is often challenging, as they may be deployed in remote regions where existing backhaul infrastructure is expensive or absent. With the commercial and industrial success of Unmanned Aircraft Systems (UAS), there is understandable interest in using UASs for delay tolerant data collection from 802.15.4 IoT sensor networks. In this study, we investigate how to optimize 802.15.4 networks for aerial data collection, which, unlike other wireless standards, has not received rigorous evaluation for three-dimensional aerial communication. We analyze experimental measurements from an outdoor aerial testbed, examining how factors, such as antenna orientation, altitude, antenna placement, and obstruction, affect signal strength and packet reception rate. In our analysis, we model and predict the quality of service for aerial data collection, based on these network configuration variables, and contrast that with the Received Signal Strength Indication (RSSI)—a commonly used signal strength metric. We find that network configuration plays a significant role in network quality, which RSSI, a mediator variable, struggles to account for in the presence of high packet loss. We conclude with a discussion of strategies for optimizing sensor network configuration for aerial data collection, in light of our results.

## 1. Introduction

Unmanned Aircraft Systems (UAS) are a promising technology for data collection from outdoor sensor networks. Environmental and agricultural applications may not have access to existing Internet backhauls for data delivery because low population densities in rural areas are unlikely to provide economic incentive for cellular providers to serve these remote areas. As these types of applications may be in difficult or inaccessible terrain that span large geographic areas, manual “sneaker-net” data collection can be dangerous and labor intensive [1].

UASs are a potential data collection alternative. To service disconnected regions, UASs are deployed as aerial network relay nodes [2,3,4,5] or as data mules [6,7]. In the event of a disruption to an existing network, such as damage to relay towers during a storm, UASs can mend network fragmentation [8,9,10], acting as temporary relays or Delay Tolerant Network (DTN) nodes. UASs can likewise supplement existing communication infrastructure, in vehicular networks [11] for example, as well as rural applications in environmental monitoring [12,13] and precision agriculture [14,15]. To adequately utilize UASs for these types of applications, network planners must understand how best to optimize connectivity so that data is successfully delivered.

In this study, we examine the IEEE 802.15.4 Low-Rate Wireless Personal Area Networks (LR-WPANs) standard [16] and its use in aerial vehicles. Unlike the 802.11 standard, the 802.15.4 radio standard emphasizes energy performance over data rates. 802.15.4, as well as derivative standards, such as Zigbee [17], WirelessHART [18], and Thread [19], are widely used for applications requiring low-rate, low-power communication, such as those utilizing Internet of Things (IoT) sensors in rural outdoor networks. The interaction between these transmission standards and aerial systems is not well understood, but is essential to the feasibility of aerial data collection for a variety of applications, including disaster management, environmental monitoring and precision agriculture [20,21,22,23].

We identify elements critical to successful data collection from an 802.15.4 2.4 GHz network using a moving quad-copter. Our work provides three key contributions: (1) We conduct performance measurements of the Received Signal Strength Indication (RSSI) and Packet Reception Rate (PRR) by evaluating the impact of variables, such as altitude, displacement, antenna orientation, obstruction, transmission rate, and transmitter elevation. (2) We use the results to model the independent impact that each variable has on RSSI and PRR. (3) We show how network configuration can be used to model and predict network performance.

Our work suggests that optimal network performance in an outdoor rural 802.15.4 2.4 GHz network can be achieved by flying the UAS at altitudes of approximately 45 m with the receiver mounted parallel to the ground. The orientation of the transmitter does not have a significant impact on reception. Further, we find that RSSI is a poor indicator of overall network performance.

## 2. Related Work

Numerous past studies have examined 802.15.4 performance in two-dimensional space. The work by Petrova et al. [24] offered insight on 802.15.4 indoor and outdoor performance in terms of RSSI and packet error rate as well as the impact of coexistence with 802.11. Similarly, Hara et al. [25] examined 802.15.4 propagation characteristics, while Miluzzo et al. [26] examined the aspects of person-to-person communication over 802.15.4. However, these two-dimensional studies on 802.15.4 emphasize network characteristics, such as throughput and RSSI, without addressing reception rates or the additional challenges of communicating in three dimensions.

While work on 802.15.4 in three-dimensional space is limited, some previous work examines 802.11 2.4 GHz performance in three dimensions. Both Asadpour et al. [27] and Lima et al. [28] revealed that the high mobility of UASs results in poor network performance for 802.11. Additionally, Cheng et al. [29] and Yanmaz et al. [30] showed that, due to the toroidal radiation patterns in consumer omni-directional dipole antennas, signal quality can be strongly affected by antenna orientation for 802.11 devices in three-dimensional space. Given that 802.11 performance is heavily impacted when used in three-dimensional highly mobile scenarios, there is reason to believe that 802.15.4 will similarly be affected and the factors impacting performance should be studied.

Experimental research on 802.15.4 for UASs is largely preliminary. Lymberopoulos et al. [31] found that 802.15.4 devices are sensitive to antenna orientation in three-dimensional space. However, their analysis is based on data collected from stationary sensors within 3 m of each other. The target scenarios of UAS-based data collection require far greater total distances (upwards of a hundred meters) using a highly mobile UAS as a receiver. In addition, Martinez et al. [32] provided a simulation of a large sensor network with data collected by a UAS. In [33], our preliminary analysis revealed that such simulated assumptions are not matched by empirical measurements. In this paper, we expand our analysis and model how network configuration predicts network performance. This project is, to our knowledge, the first performance measurement study of 802.15.4 ground–air data collection from a UAS.

## 3. Methods

**Figure 1:**
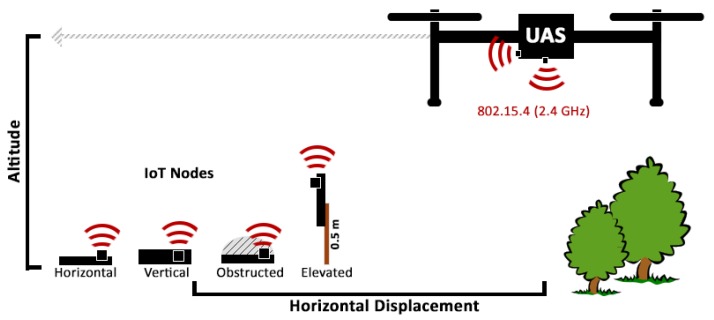
Experimental design.

Our experiment comprised approximately nine hours of experimental flight data collected from an outdoor aerial testbed, near the University of California, Santa Barbara in March 2019. We deployed four identical IoT transmitters utilizing the 802.15.4 standard, broadcasting packets at 500 ms intervals at three different locations. To collect the data, we flew a UAS at varying altitudes and distances. The experimental setup is shown in Figure 1. We conducted three repeated runs, on multiple days, at each location. Each experiment comprised approximately one hour of flight time, broken evenly across thirteen altitudes.

### 3.1. Equipment

Our equipment consisted of four transmitters on the ground, each transmitting packets received by a single UAS. To communicate, they utilized a total of six Digi WRL-15126 XBee3 RF 2.4 GHz transceivers implementing the 802.15.4 standard. The specifications for these transceivers advertise an outdoor range of 1200 m at a power of 8 dBm and a receiver sensitivity of −103 dBm [34]. We utilized the XBee3 2.4 GHz model (as opposed to 900 MHz or 868 MHz models) to make our work comparable to previous research, including work that studies 802.11 at 2.4 GHz [24,25,26,31].

#### 3.1.1. Transmitters

For transmitters, we used four XBee3 radios mounted on SparkFun XBee Explorer boards controlled by a SparkFun Teensy LC. The transmitters were powered by external USB battery packs from varying vendors via a USB-to-Serial converter on the Teensy LCs. The transmitters were configured to broadcast 23 byte packets, every 500 ms. The packets consisted of a randomly generated floating point number to simulate sensor data, as well as unique device and packet identifiers.

**Figure 2:**
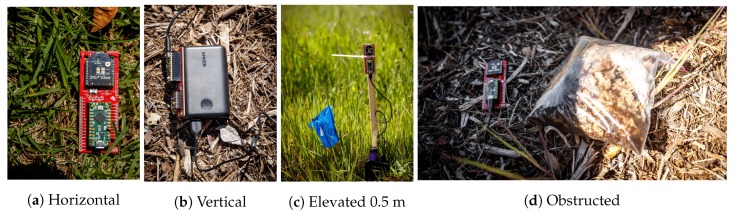
Photos of transmitter configurations.

Before each experiment, the transmitters were randomly distributed in a line, approximately 11 m apart. Placement was chosen so that there was no obstruction within the 15 cm vicinity of the transmitter. A linear configuration was chosen to ensure the UAS (flown in parallel over the transmitters) covered approximately equal horizontal displacement ranges for all transmitters. The latitude and longitude of the transmitter were recorded manually from repeated readings using a smartphone GPS. Each of the four transmitters was deployed a different configuration, as shown in Figure 2, in order to evaluate the effect of the antenna orientation, elevation, and obstruction:

- **Horizontal**: Laid flat, parallel to ground.
- **Vertical**: Placed on edge on ground.
- **Elevated**: Mounted to pole 0.5 m above ground.
- **Obstructed**: Laid flat under 1 quart of debris.

#### 3.1.2. Unmanned Aircraft System

**Figure 3:**
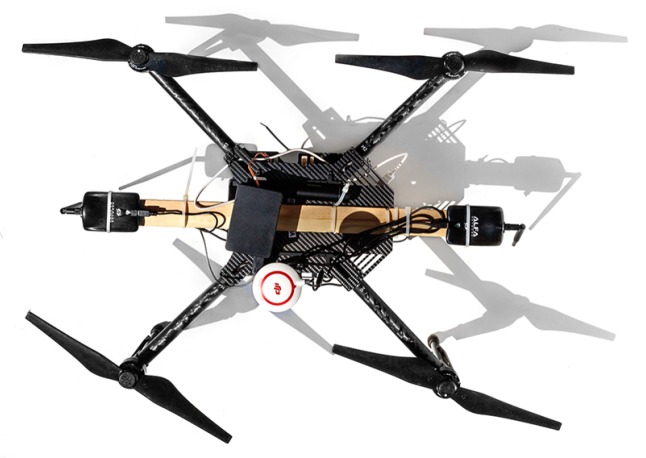
Our UAS, a DJI Matrice 100 quad-copter.

The aerial data collector was a DJI Matrice 100 quad-copter, as shown in Figure 3. Two XBee radios, set only to receive packets, were mounted to the bottom of the UAS with the *horizontal receiver* parallel to the ground and the *vertical receiver* perpendicular to the ground. The Matrice 100 communicates with a remote control at 5.725–5.825 GHz, which is outside the frequency range of the 2.4 GHz XBee nodes. The UAS was flown with no on-board camera. A Raspberry Pi 2—Model B served as an onboard computer. The two XBees forwarded packets to the Pi via USB connections. The location of the UAS was recorded from the Matrice 100 onboard GPS, sampling at a rate of 50 Hz and using a UART connection to the Pi.

We flew the UAS over the transmitters in a straight line, at an average speed of 2.2 m/s. The exact flight path and speed varied due to manual execution under varying wind condition on multiple days. Each flight consisted of 13 altitudes (in relation to ground level at the lowest transmitter) of 9 m, 12 m, 15 m, 18 m, 21 m, 24 m, 27 m, 30 m, 46 m, 61 m, 76 m, 91 m, and 122 m. Altitudes were originally chosen in feet and converted to meters for this publication, as the United States Federal Aviation Administration (FAA) regulates altitudes in feet. The maximum altitude of 122 m corresponds to the FAA max altitude limit of 400 feet. Each flight varied in total maximum horizontal displacement, but exceeded a minimum horizontal radius of 250 m from the closest transmitter to the UAS.

### 3.2. Location

The experiments were conducted at Coal Oil Point UC Reserve, a coastal grassland reserve near the university. The area is relatively flat with minor obstruction due to tall grass and bushes. In the grassland, we experimented in three locations with varying topography:

- **Road**: Transmitters were deployed along a 200 m section of a flat dirt road. The area had the lowest level of natural obstruction among the three experimental sites.
- **Grassy**: Transmitters were deployed in a field with tall grass and nearby bushes, ≈1 m tall, but with the immediate 15 cm around each transmitter unobstructed.
- **Hills**: Transmitters were deployed on the uneven terrain of hills with a shallow trench, (≈0.5 m deep), cut out by erosion. Tall grass and a denser concentration of bushes were prevalent, but the immediate 15 cm surrounding the transmitter was unobstructed.

### 3.3. Modeling RSSI

In our analysis, we analyze the distributions of RSSI by experimental variables that we hypothesize will impact RSSI (displacement, altitude, location, and transmitter/receiver configuration). To evaluate the independent influence of the variables on RSSI, we use a General Linear Model (GLM) with robust variance estimation. We set RSSI as the outcome variable and natural log of displacement, altitude (13 groups), and configuration group (8 groups) as fixed covariates while also controlling for location (3 groups). We present the results in Section 4.1 and the resulting variable estimates in Table 1.

### 3.4. Measuring Packet Reception Rate

Physical layer metrics, such as RSSI, are used to passively infer the expected performance of a network. As outdoor IoT applications are delay tolerant in nature not all metrics of always-connected wired and wireless network performance apply. Time-based metrics utilizing *latency* and *jitter* are inappropriate for this application, as the UAS might be acting as a DTN node. While *throughput* is a common metric for 802.11 networks, it may be inappropriate for 802.15.4, since typical IoT applications do not saturate a network’s bandwidth. Instead, they optimize for low-power consumption, especially in the context of outdoor sensor networks that may lack access to the power grid. We therefore examine the packet reception rate, which is the number of packets received divided by the number sent. Because each packet loss is wasted energy, PRR is a more appropriate network performance metric for this type of application.

(1) $$\mathrm{PRR}=\frac{\#\mathrm{of}\mathrm{packets}\mathrm{received}}{\mathrm{time}\;\mathrm{in}\;\mathrm{sector}*\mathrm{transmission}\;\mathrm{rate}}$$

We group the experimental data by horizontal displacement into concentric circular sectors radiating out from each transmitter (10 m wide for heat maps, and 25 m wide for models), keeping other experimental variables (altitude, receiver/transmitter configuration, and location) separated. To determine the sector into which a packet from a particular transmitter falls, we compare the UASs high frequency (50 Hz) on-board GPS with the manually recorded transmitter location. To estimate the number of packets sent by a transmitter, we calculate the product of the pre-programmed transmission rate and the time-in-sector by the UAS. We drop groupings where fewer than 5 packets were sent.

### 3.5. Predicting Packet Reception Rate

When planning a deployment, the ability to estimate the expected PRR for a network configuration and drone flight path is critical to ensuring that aerial data collection is successful. To investigate this possibility, we model the expected mean PRR based on our experimental variables.

We evaluated Poisson regression, Zero Inflated Poisson, Negative Binomial, and Zero Inflated Negative Binomial (ZINB) as possible models. By running the Vuong’s closeness test, we found that ZINB was the best fitting model, as it accounts for the over-dispersion due to high number of PRRs at zero from locations and altitudes that never receive a packet. We therefore modeled both the chances that a packet is received at all, and the estimated number of packets received. We assessed the goodness of fit for our ZINB model by Scaled Pearson Chi-Square criteria, which was close to one and by the Full Log Likelihood criteria. We also compared the observed relative frequencies of the various counts to the maximum likelihood estimates of their respective probabilities. We found that our model was a good fit for the observed data.

The input data to the model were grouped by displacement bins, as described in Section 3.4. Before analysis, we randomly divided these data into three three sets: 50% were allocated as a *training set*, 40% were allocated as a validation set, and the remaining (10%) were the *test set*. Roughly 59% of the observations received at least one packet. We used a ZINB model with the number of received packets as the outcome. We set displacement as a categorical variable with seven groups (<50 m, (50 m, 75 m], (75 m, 100 m], (100 m, 125 m], (125 m, 150 m], (150 m, 175 m], and > 175 m), altitude as a categorical variable with 11 groups (9 m, 12 m, 15 m, 18 m, 21 m, 24 m, 27 m, 30 m, 46 m, 61 m, and 76 m), and transmitter–receiver configuration with eight groups (Vertical and Horizontal Receivers paired with Horizontal, Elevated, Obstructed and Vertical Transmitters) as fixed covariates for both parts of our model. We left out location as it was not a significant variable in this model. We used a natural logarithm of sent packets as an offset in the NB part of the model and control for the number of of sent packets in the ZI part of the model. As before, we dropped bins where fewer than five packets were sent.

## 4. Results

Our results comprise nine hours of collected data, totaling 121,503 received packets, and include only the experimental portion of each flight; landing, takeoffs, and transitions between experiments are omitted. The dataset is available online [35].

### 4.1. RSSI

Past measurement studies of UAS-ground communication have focused on RSSI as a key indicator of performance [27,29,30]. RSSI is a common signal strength indicator, often the only signal metric reported by radio modules. Past work has shown that reported RSSI is proportional to the actual received signal strength [36]. We therefore begin our analysis by examining how RSSI is affected by varying experimental variables. Given the literature, we expect:

- **Altitude/Displacement**: Receivers should have the best reception in proximity to a transmitter.
- **Location**: Obstacles introduce interference, so the unobscured *road* should have the best signal.
- **Transmitter/Receiver Configurations**: Horizontal transmitters and receivers should have the best reception. Obstructions should decrease reception, while elevating equipment should increase it.

As described in Section 3.3, we model RSSI using a GLM. This allows us to examine the independent influence of our experimental variables on RSSI. We summarize the model estimates in the left part of Table 1, predicted values of RSSI with confidence intervals from the GLM. For a baseline, we set what we consider reasonable variable choices for a realistic IoT UAS collected deployment: an elevated transmitter in a location with low obstruction, flying the UAS 101–125 m away, at an altitude of 45 m above ground level, using a horizontal receiver. In our analysis, we contrast other variable choices to this baseline.

#### 4.1.1. Altitude

**Figure 4:**
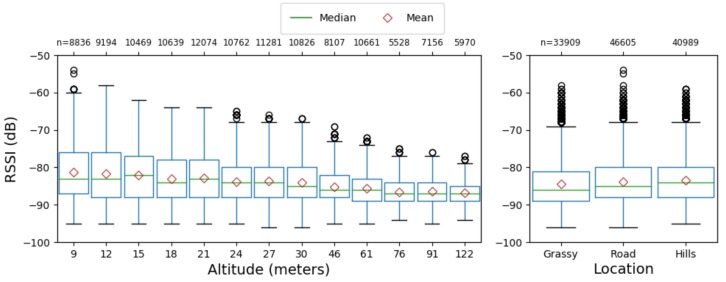
Observed RSSI Distribution. Left: grouped by altitude; Right: grouped by horizontal displacement.

We present the distribution of the reported RSSI by altitude from ground level at the location of take-off in Figure 4 (left). As only the radio module reports RSSI, the data represent only packets successfully captured and decoded by the radio module. We observe that higher altitudes have fewer high RSSI values. However, the mean and median values decrease only slightly as altitude increases.

**Figure 5:**
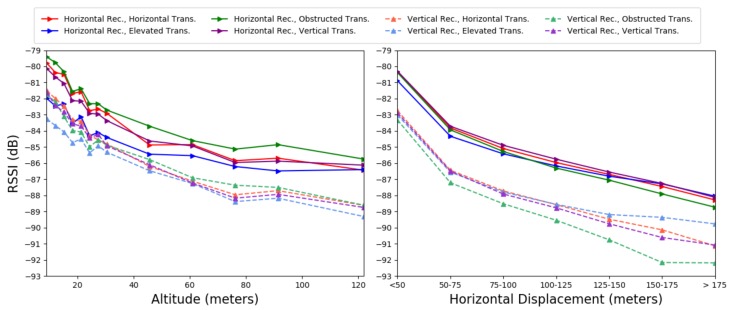
Predicted mean RSSI from GLM. Left: mean RSSI grouped by altitude; Right: mean RSSI grouped by horizontal displacement.

We examine the predicted effect of altitude on RSSI from the GLM averaging across displacements and locations, as presented in the Figure 5 (left). As expected, we find that, for all receiver/transmitter configurations, RSSI decreases with altitude; however, this mean decrease is small (<1 dB per 3 m) compared to the wide fluctuations in individual observed RSSI measurements. In Table 1, altitude changes the RSSI by a maximum of 4 dB and the trend is not monotonic.

#### 4.1.2. Location

We present the distribution of RSSI for the three locations in Figure 4 (right). The mean and median RSSI values remain similar across the three locations. However, counter to expectations, the most obstructed site (Hills) displays the highest median RSSI, and the area of least obstruction (Road) yields the greatest variance in RSSI values, although, once again, the difference in dB is small. When examining the predicted values from GLM, we observe a different pattern with the Road displaying the optimal RSSI but with 1dB of difference.

#### 4.1.3. Horizontal Displacement

Averaging across altitudes and locations, we examine the predicted impact of displacement on RSSI from the GLM. As shown in Figure 5 (right), we observe that greater horizontal displacement reduces RSSI slightly (approximately 2 dB per 30 m) for all receiver/transmitter configurations. When comparing displacements to the baseline in Table 1, we observe a 1 dB monotonic drop per 25 m displacement. Once again, we note that the mean decrease is small relative to the total observed fluctuations in individual RSSI measurements.

#### 4.1.4. Transmitter/Receiver Configurations

**Figure 6:**
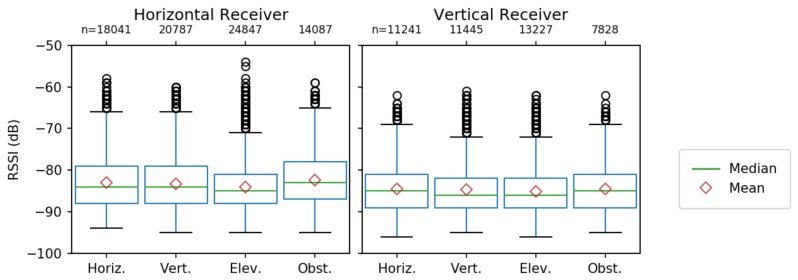
Observed RSSI distributions by configuration.

We examine the distributions for data grouped by configuration of the receiver and the transmitter. In Figure 6, the left part of the graph represents packets collected by the *horizontal receiver* (mounted on the UAS parallel to the ground), while the right shows data collected by the *vertical receiver* (mounted perpendicular to the ground). For each receiver, the data are further categorized by transmitter configuration on the ground (Horizontal, Vertical, Elevated, and Obstructed).

For the observed distributions, *transmitter* and *receiver* orientation minimally affect mean RSSI. However, *receiver* orientation has a slightly more pronounced impact. All four transmitters had lower observed mean RSSI for the *vertical receiver* than the *horizontal receiver*. These observations are consistent with the predicted values from the GLM. With regards to the effect of *horizontal displacement* on receiver/transmitter configuration in Figure 5 (right), *receiver* orientation slightly impacts RSSI, while *transmitter* orientation minimally impacts RSSI. Mirroring this result in Table 1, receiver orientation changes RSSI by approximately 3 dB, while the transmitter orientation makes little difference.

When examining the impact of *altitude* on receiver/transmitter configuration performance, we identified a more complicated interaction for the mean RSSI predicted by the GLM in Figure 5. While the horizontal *receiver* still performs better overall, the *transmitter* behaviors are contrary to expectations. The obstructed transmitter paradoxically outperforms the elevated transmitter for all altitudes.

The similarity in RSSI between transmitter configurations is contrary to expectations, especially comparing the elevated transmitter, with superior line of sight, to the obstructed transmitter, which is buried in debris. For the observed distributions in Figure 6, all transmitters broadcast at the same rate (one packet per 0.5 s), yet the *elevated* transmitter delivers a greater number of packets than the *obstructed* one (in the horizontal case, 10 k more). Therefore, the obstructed transmitter is in fact delivering fewer packets despite showing an overall better RSSI.

#### 4.1.5. Deviations from Expectations

Overall, the observed RSSI does not match the expectations stated at the start of this section. When examining mean RSSI alone, one might conclude that the experimental variables minimally impact network performance. Further, the slight improvement in RSSI due to obstructing the transmitter proves yet more confusing.

A high RSSI reflects successfully received packets, while lost packets are unaccounted for in the data. Their RSSI is never reported to the receiver module. Therefore, the mean of the received RSSI remains relatively consistent despite changes to experimental variables, especially when compared to the fluctuation in RSSI for fixed experimental variables, due to outside factors, such as external RF interference and minor changes to reception geometry due to small variations in flight paths.

Therefore, for applications involving UAS data collection on 802.15.4, RSSI alone does not provide a complete picture of network performance for our data. As noted above, while our analysis of RSSI showed little mean fluctuation between experimental configurations, our total number of received packets indicates significant differences in network performance not accounted for by RSSI. We therefore must investigate further to adequately assess the performance of our network during aerial data collection.

### 4.2. PRR by Altitude and Displacement

**Figure 7:**
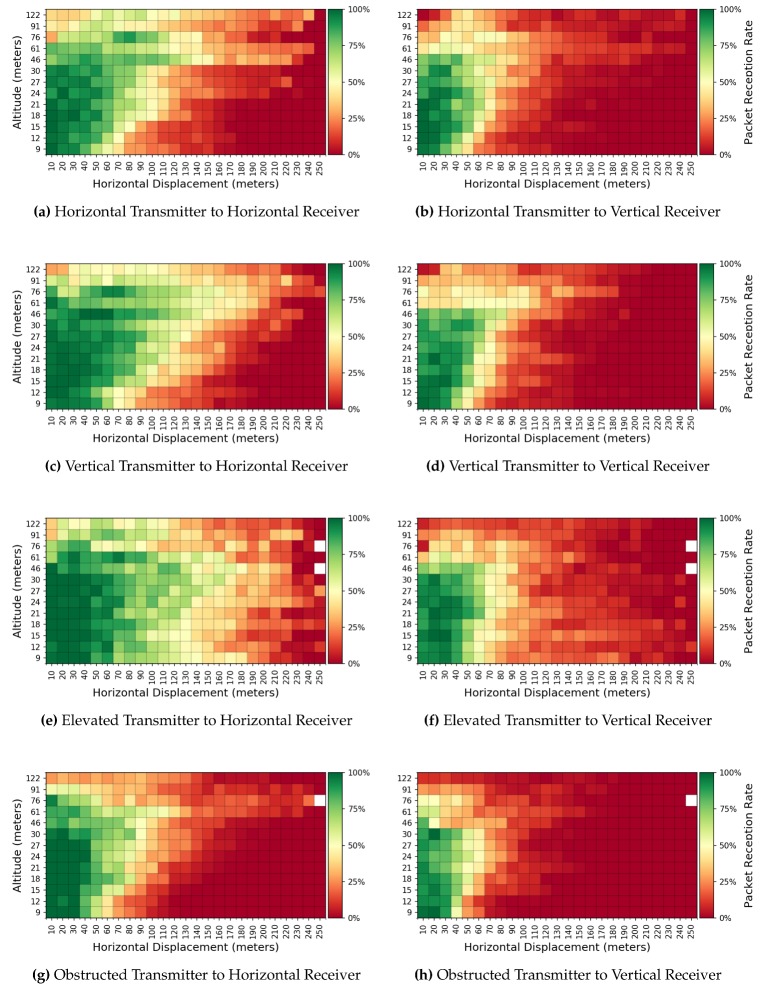
Observed packet reception rates grouped by altitude and 10 m displacements from transmitter. Cells with fewer than five sent packets are left blank.

We examine the underlying network performance via the observed PRR. As introduced in Section 3.4, we grouped the measurements into 10m concentric circular sectors radiating from each transmitter, resulting in 29,161 grouped measurements. The observed PRR (averaged across runs and locations) is presented in Figure 7 with each heat map representing a transmitter/receiver configuration.

As expected, close proximity to the transmitter, in terms of both altitude and horizontal displacement, leads to a higher rate of received packets. A greater horizontal displacement from the transmitter has greater packet loss. When examining PRR, most configurations demonstrate a sharp drop in RSSI for distances over 150 m, while the mean RSSI displays a small decrease. While lower altitudes produce a better PRR at lower horizontal displacements, altitudes between 46 and 76 m show improved performance at greater displacements. Thus, a UAS need not fly low to the ground, where it is more likely to hit obstacles, in order to optimize data collection.

Likewise, *receiver* orientation noticeably impacts loss. For all transmitters, the *vertical receiver*’s PRR is the worst at higher altitudes and displacements. According to Figure 7e,f, the PRR is best for lower altitudes, where elevating the transmitter 0.5 m off the ground overcomes ground obstacles (such as tall grass) to establish line-of-sight with the UAS. Furthermore, Figure 7g,h show that, while the *obstructed transmitter* maintains a high PRR when the UAS is in proximity, the maximum horizontal displacement at which the UAS has good reception is lower than other configurations, especially at lower altitudes. This behavior is masked when examining RSSI.

### 4.3. Optimizing PRR

We model how experimental variables impact PRR using a ZINB model, described in Section 3.5. The input data consisted of 7906 observations (divided by displacement bins of 25 m), and divided into a training set (3910 observations), validation set (3166 observations), and test set (830 observations). The model’s estimated PRRs are presented on the right side of Table 1.

Using this model, we can predict (for a given altitude, horizontal displacement, and receiver/transmitter configuration) the expected mean PRR. This serves to help identify the conditions under which a packet of data can be delivered to the drone, as might be required, for example, when planning a deployment.

**Figure 8:**
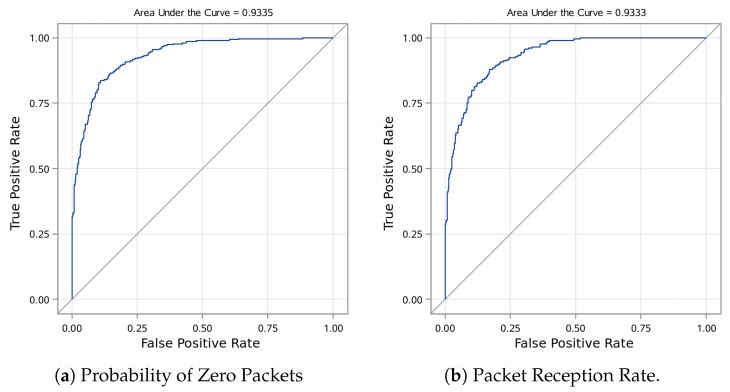
ROC curves for ZINB model estimates of test set.

We can convert the ZINB model, which predicts PRR, into a binary classifier, predicting when at least one packet will be received, by specifying a threshold below which we expect the packet to be lost. This can be done in two ways. In Figure 8, we present a Receiver Operating Characteristic (ROC) graph from the *test set* displaying the possible true and false positive rates based on threshold choices. Figure 8a employs the ZI part of the model to calculate the probability that zero packets are received and shows the corresponding ROC. This method does not incorporate the number of sent packets, and could be sensitive to transmission rates. Figure 8b first calculates predicted PRR, incorporating number of sent packets in the modeling, and then shows the resulting ROC. For example, picking a PRR threshold of 40% for the *test set*, the resulting binary classifier correctly identifies 86.9% of the observations with a specificity of 86.2% and recall of 87.5%.

#### 4.3.1. Altitude

**Figure 9:**
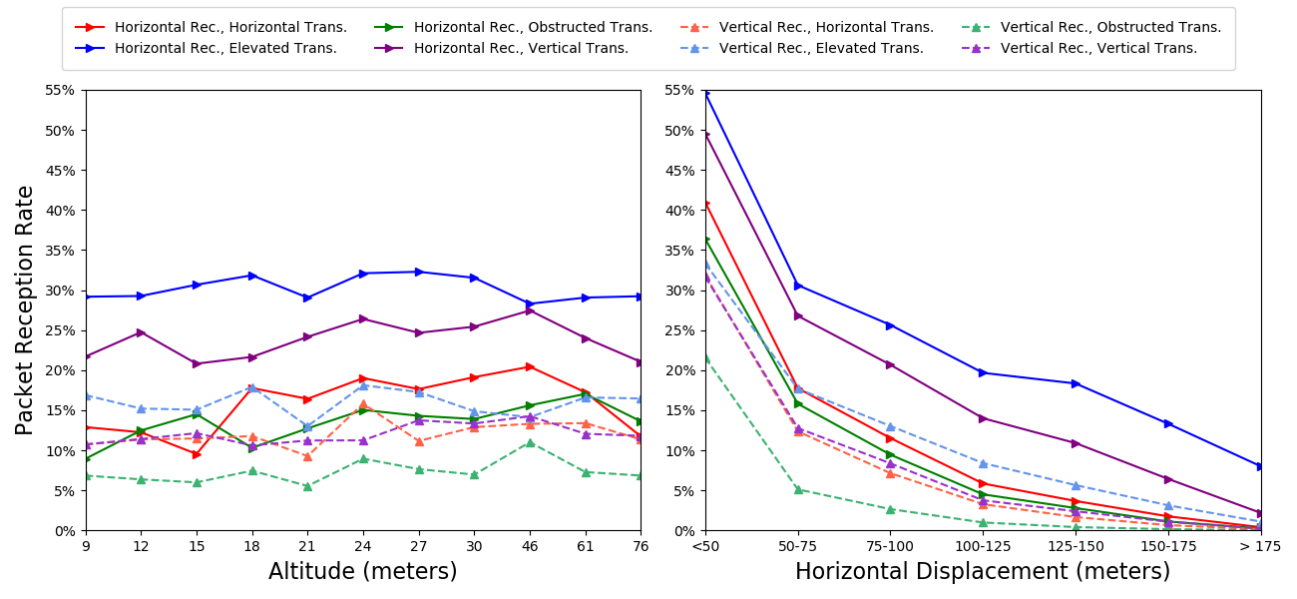
Predicted mean PRR from ZINB model for each receiver/transmitter configuration. Left: grouped by altitude; Right: grouped by horizontal displacement.

Based on the ZINB model of our training set, we examine the effect of altitude on the PRR. Figure 9 (left) shows the impact of altitude by group (averaging across displacements). For most transmitter/receiver configurations, PRR peaks at 46 m altitude. As observed in the Figure 7 heat maps, the impact of altitude seems tied to displacement. When fixing a displacement, as we do in Table 1, the altitude minimally effects PRR with single percent fluctuations until altitude exceeds 61 m.

#### 4.3.2. Horizontal Displacement

We similarly use the ZINB model of our training set to examine the effect of horizontal displacement on the PRR. In Figure 9 (right), we show the impact of displacement by group (averaging across all altitudes). Here, the effect is more linear, with displacement causing a monotonic drop in PRR until no packet is received. When comparing to our baseline, in Table 1, we observe a nearly 5% drop in PRR per 25 m, after 50 m.

#### 4.3.3. Transmitter/Receiver Configuration

As expected, the elevated transmitter has a significantly better PRR across all altitudes and displacements, while the obstructed transmitter has the worst. The horizontal and vertical transmitters behave similarly. The horizontal receiver shows better PRR for all groups. When compared to our baseline in Table 1, a horizontal receiver gives a 3–8% increase in PRR. We similarly observe that obstruction causes a 2–4% decrease in PRR compared to a horizontal transmitter, while elevating the transmitter gives a 5% boost but only in the case of a horizontal receiver.

#### 4.3.4. Predicting PRR from RSSI

Predicting PRR from RSSI can be difficult because RSSI remains unreported when no packets are received (i.e., the PRR is zero). The ZINB model comprises two parts, a logistic regression model predicting the probability any packets are received and a negative binomial model predicting a PRR given that a packet is likely to be received.

**Figure 10:**
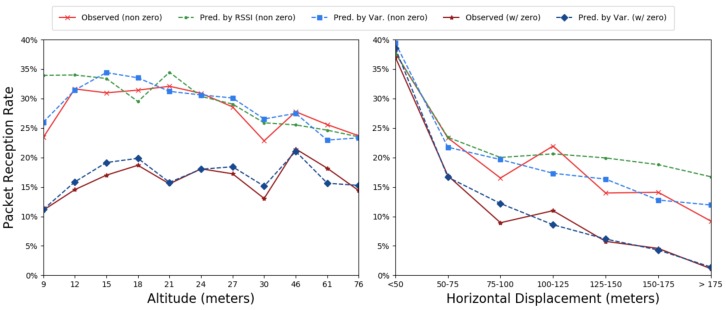
Accuracy of predicted mean PRR. (**a**) grouped by altitude; (**b**) grouped by horizontal displacement.

To examine whether RSSI, acting as a mediator variable, can be used to model and predict PRR, we used RSSI to train a negative binomial model on our *training set*. To properly compare it, we also reran the negative binomial portion of the model based on experimental variables for our *training set*, dropping PRRs of zero. We evaluate our model on the *test set*, without PRRs of zero. Figure 10 shows the results of the two models. We compare the predictions of those models to the observed PRR in the *test set* (including and not including PRRs of zero), as well as the result of the full ZINB model, which predicts using experimental variables.

When we ignore PRRs of zero and average across other variables, RSSI closely predicts PRR. However, the model that is based on experimental variables, rather than RSSI, is still a better fit to the observed data. While inferring PRR, in order to gauge how many re-transmissions may be required for successful data delivery, may be useful, doing so fails to predict whether packets will be received in the first place. When we compare the RSSI prediction to the observed readings that include PRRs of zero, RSSI over-predicts the real PRR by more than double. In contrast, the ZINB model, trained to predict total packet loss, is a good fit.

## 5. Discussion

Our measurement study provides insight into optimizing an IoT deployment for aerial data collection by a UAS. While existing literature focuses primarily on mean RSSI as a principle indicator for an aerial network’s performance, our analysis reveals that RSSI inadequately reflects the network behavior. Because radio modules report RSSI only when a packet is successfully delivered, RSSI fails to capture steep drops in PRR. Instead, in open spaces without strong sources of interference, the geography of a network, such as distance from node and mounting of transmitters, can be used to estimate the expected performance. In this section, we discuss how, based on our results, one can optimize such an 802.15.4 network for aerial data collection.

### 5.1. Effective Reception Range

During initial planning of this measurement campaign, we expected to observe connectivity at distances far greater than those measured. Digi advertises an effective total distance of 1200 m for the XBee3, with the caveat that “[a]ctual range will vary based on transmitting power, orientation of transmitter and receiver, height of transmitting antenna, height of receiving antenna, weather conditions, interference sources in the area, and terrain between receiver and transmitter” [34]. However, in near optimal real-world conditions, we only successfully captured packets at a maximum total distance of 297 m in our experiments. When accounting for the UASs altitude, this corresponds to a 278 m maximum horizontal displacement from the transmitter, but the UAS was highly unlikely to receive a packet at this displacement.

### 5.2. Optimal Altitude

Although low altitudes generally improve PRR and RSSI, higher altitudes provide better connectivity at greater horizontal displacements. We theorize that higher altitudes provide a steeper angle between the UAS and transmitter, which reduces signal blockage from obstacles, such as trees and bushes. An altitude of 46 m provides the best overall reception. This is fortunate as a high altitude allows a UAS to clear most trees and ground obstacles.

### 5.3. Optimal Antenna Orientation

Previous work investigates how radiation patterns cause antennae orientations to affect signal quality. In our work, the antenna orientation of the *transmitter* did not significantly impact performance, while the orientation of the *receiver* had a far greater impact, with the vertically mounted receiver performing substantially worse than the horizontally mounted one.

In our case, this may indicate that signal radiation is a smaller factor than minor fluctuations in line-of-sight. Unlike past experiments that employed a 1.1 inch straight wire as an antenna for a 802.15.4 2.4 GHz CC2420 transmitter [31], our setup employed a coiled embedded antenna directly on the comparatively smaller XBee. While Lymberopoulos et al. [31] studied distances of <10 m, our work includes signals at distances of >250 m where the topography and antennae geometry might reduce impact of orientation.

The better performance compared to the vertically mounted receiver could be due to the superior line of sight to the horizontally mounted receiver. The *horizontal receiver* was parallel to ground at all times, while the *vertical receiver*’s own body may have blocked the signal from unfavorable angles.

In further consideration of transmitter orientation, aerial networks might serve as auxiliary modes of connection to on-the-ground infrastructure. For example, cluster heads may use the same 802.15.4 radio to communicate with other nodes and the UAS. In such a case, tailoring ground transmitters to communicate with one another without taking communication with the UAS into account would be optimal.

### 5.4. Elevating Transmitters

In IoT deployments where elevating the transmitter is possible, it is advisable to do so. While the *elevated transmitter* had a very similar RSSI performance to the *horizontal transmitter* on the ground, the PRR of the elevated transmitter was much improved, especially for lower UAS altitudes. The transmitter cleared much of the ground-level obstruction and attained better line-of-sight to the UAS when elevated even half a meter above the ground. The optimal height for a transmitter may depend on the specific local geography.

### 5.5. Obstruction

Sensor nodes are frequently deployed in the field with little protection from extreme weather. Nodes and antennas can be affected by various obstructions, including dirt from rain or wind. While obstruction minimally affects reported RSSI, it significantly impacts PRR. Buried sensors communicate at a high loss rate. However, most packets are lost at greater distances, wasting transmission power. If the IoT device relies on solar power, which may likewise become obstructed, this exacerbates the issue.

### 5.6. Transmission Rate Selection

IoT deployments typically minimize the transmission rate in order to save power. However, we found that achieving this may be difficult when performing aerial data collection with a UAS, since the flight time on consumer multi-copters as of spring 2019 is approximately 20 min (1200 s) per battery. As flight speed correlates with successful data capture at a particular data transmission rate, multi-copter battery capacity constrains viable transmission rates. Fixed-wing aircraft are similarly constrained as they have a minimum flight speed of >10 m/s.

We flew the quad-copter at an average speed of 2.2 m/s (5 mph). On a single battery, at this speed, the UAS could cover 2.6 km. This is already a relatively small coverage area when accounting for a round trip flight—approaching the lowest feasible flight speed for UAS-based data collection.

At our low flight speed, both 500 ms and 1 s inter-packet transmission rates produced similar RSSI and PRR values. While we attempted slower transmission rates, we received too few packets for a meaningful analysis. At one packet per 15 s, we received an average of only 258 packets per transmitter–receiver pair across all locations, altitudes, and repeated trials. For one packet per minute, the average per transmitter-receiver pair was only 127 packets. Given the experimental results, determining the max flight speed that guaranteed delivery at low transmission rates is difficult, as the data is sparse.

This is a potential complication for aerial collection, as high transmission rates would correspond to increased power consumption. Strategies such as pre-scheduling collection windows or a signaling mechanism, during which the transmitters increase rate, could mitigate this issue.

## 6. Conclusions

In this study, we reviewed the effects of altitude, antenna orientation, obstruction, antenna elevation, and transmission rate on RSSI and PRR of a 802.15.4 2.4 GHz IoT network. We found that RSSI is a weak indicator of network performance for aerial data collection of an IoT network, as it poorly reflects high levels of packet loss. When examining reception rate, our experimental variables had far greater variability than RSSI showed.

An 802.15.4 network optimized for ground based communication may not be suitable for aerial data collection without modification and consideration of a variety of factors, such as device placement, altitude of collection, transmission rate, and resilience to obstruction. Our work provides a model for making informed choices about these factors.

As future work, we will investigate the performance of aerial collection using 802.15.4 in other environments, such as urban IoT networks, and for other models of sensors, including those with external mono-pole antenna. We will also explore the automatic mapping of IoT nodes, as well as ascertaining the health of IoT nodes from network characteristics, for example detecting transmitters obstructed by debris. We hope to apply these efforts to better understand the applicability of UAS networks for environmental monitoring and post-disaster recovery.

## Figures and Tables

**Table 1:** Estimates by parameter for GLM and ZINB Models.

		RSSI GLM Model			PRR ZINB Model		
Parameter		Estimated Mean RSSI	Mean Confidence Interval		Estimated Mean PRR	Mean Confidence Interval	
Baseline	Rec. Horiz. Trans. Elev. Alt. 150 ft D. 101–125 m Loc. Road	−87.2114	−87.1066	−87.3162	0.204	0.1777	0.2341
Rec. H.	Trans. Horiz.	−86.5816	−86.4676	−86.6956	0.1528	0.1319	0.1769
	Trans. Obs.	−86.8098	−86.6867	−86.933	0.1369	0.1175	0.1595
	Trans. Vert.	−86.5092	−86.3997	−86.6187	0.1865	0.1625	0.214
Rec. V.	Trans. Horiz.	−89.2424	−89.1179	−89.3668	0.1218	0.1046	0.1418
	Trans. Elev.	−89.6226	−89.503	−89.7422	0.125	0.1082	0.1445
	Trans. Obs.	−90.155	−90.0124	−90.2977	0.0877	0.0746	0.1032
	Trans. Vert.	−89.2804	−89.1584	−89.4023	0.1203	0.1038	0.1395
Altitude	9 m	−84.1888	−84.0457	−84.3319	0.1859	0.1626	0.2126
	12 m	−84.2479	−84.107	−84.3889	0.2025	0.1779	0.2304
	15 m	−84.5907	−84.4674	−84.714	0.2147	0.1884	0.2446
	18 m	−85.2649	−85.1463	−85.3836	0.2117	0.1859	0.2411
	21 m	−85.1696	−85.0613	−85.2778	0.2025	0.1781	0.2302
	24 m	−85.8722	−85.7614	−85.983	0.2234	0.1971	0.2533
	27 m	−85.6163	−85.5125	−85.7201	0.21	0.1854	0.2378
	30 m	−85.7763	−85.6692	−85.8834	0.213	0.1873	0.2422
	61 m	−86.8529	−86.7577	−86.9481	0.1897	0.1676	0.2147
	76 m	−88.1146	−88.006	−88.2232	0.1563	0.1354	0.1805
	91 m	−87.4706	−87.3676	−87.5736	-	-	-
	122 m	−87.7332	−87.6294	−87.837	-	-	-
Displacement	<50 m	−82.1024	−81.9858	−82.2189	0.5518	0.4923	0.6185
	50–75 m	−85.3911	−85.2856	−85.4966	0.3116	0.2746	0.3535
	76–100 m	−86.4356	−86.331	−86.5402	0.258	0.2276	0.2925
	126–150 m	−87.8248	−87.7193	−87.9303	0.1984	0.1715	0.2295
	151–175 m	−88.3346	−88.2282	−88.441	0.1535	0.1302	0.1809
	>175 m	−88.9355	−88.8277	−89.0434	0.1271	0.1036	0.1559
Loc.	Grassy	−88.2025	−88.0827	−88.3222	-	-	-
	Hills	−87.2797	−87.1742	−87.3853	-	-	-

This table shows the mean estimates for possible experimental variable choices for the two models, using the top row’s variable choices as a baseline. Each subsequent row reflects the effect of changing that one variable while keeping all other variables to their baseline settings (Horizontal Receiver, Elevated Transmitter, Altitude of 46 m, Displacement of 101–125 m, at the Road location). Altitudes > 76 m and locations are not in the PRR ZINB model. All *p*-values for reported data are <0.0001.

## Abbreviations

The following abbreviations are used in this manuscript:

UAS	Unmanned Aircraft System
GLM	General Linear Model
DTN	Delay Tolerant Network
IOT	Internet of Things
RSSI	Received Signal Strength Indication
FAA	United States Federal Aviation Administration
PRR	Packet Reception Rate
ZINB	Zero Inflated Negative Binomial Model
ROC	Receiver Operating Characteristic
